# TARO: tree-aggregated factor regression for microbiome data integration

**DOI:** 10.1093/bioinformatics/btae321

**Published:** 2024-05-24

**Authors:** Aditya K Mishra, Iqbal Mahmud, Philip L Lorenzi, Robert R Jenq, Jennifer A Wargo, Nadim J Ajami, Christine B Peterson

**Affiliations:** Department of Genomic Medicine, The University of Texas MD Anderson Cancer Center, Houston, TX 77030, United States; Platform for Innovative Microbiome and Translational Research (PRIME-TR), The University of Texas MD Anderson Cancer Center, Houston, TX 77030, United States; Department of Bioinformatics and Computational Biology, The University of Texas MD Anderson Cancer Center, Houston, TX 77030, United States; Department of Bioinformatics and Computational Biology, The University of Texas MD Anderson Cancer Center, Houston, TX 77030, United States; Department of Genomic Medicine, The University of Texas MD Anderson Cancer Center, Houston, TX 77030, United States; Platform for Innovative Microbiome and Translational Research (PRIME-TR), The University of Texas MD Anderson Cancer Center, Houston, TX 77030, United States; Department of Genomic Medicine, The University of Texas MD Anderson Cancer Center, Houston, TX 77030, United States; Platform for Innovative Microbiome and Translational Research (PRIME-TR), The University of Texas MD Anderson Cancer Center, Houston, TX 77030, United States; Department of Surgical Oncology, The University of Texas MD Anderson Cancer Center, Houston, TX 77030, United States; Department of Genomic Medicine, The University of Texas MD Anderson Cancer Center, Houston, TX 77030, United States; Platform for Innovative Microbiome and Translational Research (PRIME-TR), The University of Texas MD Anderson Cancer Center, Houston, TX 77030, United States; Platform for Innovative Microbiome and Translational Research (PRIME-TR), The University of Texas MD Anderson Cancer Center, Houston, TX 77030, United States; Department of Biostatistics, The University of Texas MD Anderson Cancer Center, Houston, TX 77030, United States

## Abstract

**Motivation:**

Although the human microbiome plays a key role in health and disease, the biological mechanisms underlying the interaction between the microbiome and its host are incompletely understood. Integration with other molecular profiling data offers an opportunity to characterize the role of the microbiome and elucidate therapeutic targets. However, this remains challenging to the high dimensionality, compositionality, and rare features found in microbiome profiling data. These challenges necessitate the use of methods that can achieve structured sparsity in learning cross-platform association patterns.

**Results:**

We propose Tree-Aggregated factor RegressiOn (TARO) for the integration of microbiome and metabolomic data. We leverage information on the taxonomic tree structure to flexibly aggregate rare features. We demonstrate through simulation studies that TARO accurately recovers a low-rank coefficient matrix and identifies relevant features. We applied TARO to microbiome and metabolomic profiles gathered from subjects being screened for colorectal cancer to understand how gut microrganisms shape intestinal metabolite abundances.

**Availability and implementation:**

The R package TARO implementing the proposed methods is available online at https://github.com/amishra-stats/taro-package.

## 1 Introduction

The human microbiome consists of a diverse community of microorganisms, including bacteria, fungi, and viruses, that populate various sites in the body. The microbiome plays a key role in many normal biological processes in the host including digestion and immune regulation, while dysbiosis, or disruption of healthy microbiome composition, has been linked to disease risk across a range of conditions, including heart disease, diabetes, and cancer (Hou *et al.* 2022). Although mechanisms of host-microbiome interaction remain incompletely understood, one avenue for the influence of the microbiome on host disease processes is through the production of metabolites (Cullin *et al.* 2021). Characterizing the influence of the microbiome on the metabolome requires integrative analysis of these high-throughput data types. However, this task is challenging for several reasons: both microbiome and metabolite data are high dimensional, with thousands of features measured in each sample; microbiome data are compositional, which means that each sample has a fixed sum constraint; and microbiome data are zero-inflated, which means that a feature observed in one sample is often not observed in other samples, resulting in rare features with a large number of observed zero values.

Various methods have been proposed for cross-platform integration of microbiome and metabolomics data including correlation and network inference approaches. A naïve method popular in practice is to test for pairwise associations between individual features using Pearson’s or Spearman’s correlation; however, this approach creates a high multiple testing burden. Classical multivariate methods, such as canonical correlation analysis (CCA) and co-inertia analysis (CIA), are attractive alternatives but require that the number of samples *n* is larger than the number of variables. Given the high dimensionality of datasets in the high-throughput era, sparse versions of CCA and CIA have been developed to resolve this limitation (Witten and Tibshirani 2009, Min *et al.* 2019). Network inference methods based on the graphical modeling framework have also been proposed as an approach for the integration of microbiome data with high-dimensional covariates (Yang *et al.* 2017, Osborne *et al.* 2022); an advantage of these methods is that they aim to capture direct associations by focusing on conditional, rather than marginal, correlations. However, none of these methods directly handle the challenge of rare features.

Here, we frame the challenge of integrating microbiome and metabolite data as a factor regression model, with the microbiome profiles as the predictor and metabolite profiles as the response. We propose Tree-Aggregated factor RegressiOn (TARO), building on the reduced-rank regression framework to enable the discovery of interpretable latent factors with flexible aggregation of rare features. Our proposed approach leverages information on the taxonomic tree to enable aggregation of features in a data-adaptive manner, collapsing rare features into aggregated features that are less zero-inflated. Our proposed method offers a comprehensive solution to the challenges of high dimensionality, compositionality, and rare features. In Section 2, we provide a description of the proposed model and estimation procedure. In Section 3, we compare the performance of TARO to alternative methods through simulation studies and apply TARO to integrate microbiome and metabolomics data from a real-world study on colorectal cancer (Yachida *et al.* 2019). Finally, we conclude with a discussion in Section 4.

## 2 Materials and methods

### 2.1 TARO model

Our proposed method builds on the multivariate regression framework to relate the microbiome and metabolomic profiling data. We assume that the observed microbiome data consists of abundances for *p* features across *n* samples. The features may correspond to taxonomic units quantified through marker gene sequencing, such as amplicon sequence variants (ASVs) or operational taxonomic units, or more generally to any functional or taxonomic read-outs. We denote the microbial abundance table as $W={\left[w_{ij}\right]}_{n\times p}={\left[w_{1},\ldots ,w_{n}\right]}^{T}$. Importantly, due to the methods employed for the generation and processing of the genomic sequences, the observed data are compositional; this means that the observed counts can only be interpreted on a relative scale (Gloor *et al.* 2017). Regression models with microbiome features as the predictor typically rely on data transformations to address this challenge (Aitchison and Bacon-Shone 1984). Here, we first apply total sum scaling (TSS), which entails dividing each count *w_ij_* by the total number of counts for its sample $\sum_{j}w_{ij}$. Recent work has shown that TSS scaling, although quite simple, tends to perform better in practice than other normalization methods (Mallick *et al.* 2021). This results in a relative abundance matrix $\tilde{W}={\left[{\tilde{w}}_{ij}\right]}_{n\times p}={\left[{\tilde{w}}_{1},\ldots ,{\tilde{w}}_{n}\right]}^{T}$ such that $\sum_{j}{\tilde{w}}_{ij}=1$ for i=1,…,n. We then apply a log transform to obtain the matrix $X={\left[x_{ij}\right]}_{n\times p}$ where $x_{ij}=\text{log}({\tilde{w}}_{ij})$. To avoid numerical issues with exact zeros, we add a pseudocount of 1 to the count matrix W prior to scaling. Importantly, the resulting *p* features are not independent, as there are only *p–*1 degrees of freedom due to the original sum constraint.

We, now, consider the formulation of the regression model relating the microbial profiling data X to the metabolite abundances. We let $Y={\left[y_{ik}\right]}_{n\times q}={\left[y_{1},\ldots ,y_{n}\right]}^{T}\in R^{n\times q}$ represent the metabolite abundances; since metabolomic data are often highly skewed, the *y_ik_* may be taken as the log-transformed concentration values. The metabolite abundances can be modeled as a function of a set of covariates $Z_{n\times m}$ and the microbiome profiles $X_{n\times p}$ via the multivariate regression:

(1) Y=Zβ+XC+E,

where $\beta_{m\times q}$ represents the matrix of effects of the clinical covariates on the metabolites, $C_{p\times q}$ is the matrix of effects of the microbiome features on the metabolites, and $E_{n\times q}={\left[e_{ik}\right]}_{n\times q}$ is the error matrix. We include an intercept in the model by setting the first column of Z to be $1_{n}$. The remaining columns correspond to clinical variables we wish to include as adjusters in the model and are not subject to selection. We, therefore, do not impose any regularization on β.

The novelty of the TARO method lies in how we estimate the coefficient matrix **C** to handle the compositionality of the microbiome profiles, aggregate rare features, and achieve sparsity. Due to the fixed sum constraint within each sample, the *p* microbiome predictor variables are not independent; this means that an additional constraint is needed to ensure identifiability of the coefficient matrix **C**. Following Lin *et al.* (2014), we incorporate a zero-sum constraint on each row of C:

$$\sum_{j=1}^{p}c_{jk}=0\;\;\text{for}\;\;k=1,\ldots ,q,$$

which can be written in matrix form as $1_{p}^{T}C=0_{q}$.

Next, we consider how to aggregate rare features; this is a critical challenge in microbiome data analysis since the observed fine-resolution microbiome features typically include features that are nonzero in only a few samples. Many existing approaches for microbiome regression collapse the observed features to a higher taxonomic rank, typically the genus level (Lin *et al.* 2014, Liu *et al.* 2022). For example, the counts for ASV1 and ASV2 could be summed to obtain the abundance for their parent genus in the taxonomic tree T. This can be carried out to obtain abundances for any internal node in the tree. Suppose we obtain a new aggregated feature for the *i*th subject $x_{i,a}=x_{i,1}+x_{i,2}+\cdots x_{i,p_{a}}$, where *p_a_* denotes the number of leaf nodes descending from the parent node *a*. As noted in Yan and Bien (2021), $x_{i,a}\beta =(x_{i,1}+x_{i,2}+\cdots x_{i,p_{a}})\beta =x_{i,1}\beta +x_{i,2}\beta +\cdots x_{i,p_{a}}\beta$. Effectively, this means that learning a model where some features have exactly equal coefficients *β* corresponds to aggregating the original features into less zero-inflated groupings. Here, we build on the work of Yan and Bien (2021) and Bien *et al.* (2021) to allow flexible estimation of the microbiome coefficients, to allow grouping of rare features when the data supports their having equivalent effects on the outcome. Using the TARO method, we learn the optimal level of aggregation from the data. However, in practice, we expect that aggregation will mostly occur over lower levels of the tree (i.e. collapsing to the genus or family level), both because there are more rare features at finer levels of resolution and because higher taxonomic ranks such as phylum, class, or order may be too heterogeneous for the data to support a shared coefficient value.

Following Yan and Bien (2021), we denote the nodes of the taxonomic tree T using an index set u∈{1,…,|T|−1}, excluding the root node, and let $A_{p\times (|T|-1)}=[a_{ju}]$ indicate the ancestry of each observed feature, where the entry *a_ju_* = 1 if microbiome feature *j* belongs to the set of leaves descending from node *u* or, for leaf nodes, if *j *=* u*. We set *a_ju_* = 0 otherwise. To enable flexible feature aggregation, we rewrite the coefficient matrix C as follows:

C=A×Γ,

where $\Gamma_{(|T|-1)\times q}$. This reparameterization results aggregated features as $\tilde{X}=XA\in R^{n\times (|T|-1)}$. We can then expand the model (1) as:

(2) $$\begin{array}{c} Y=Z\beta +XC+E,\;s.t.\;\;C=A\times \Gamma \text{ and }1_{p}^{T}C=0_{q}\\ Y=Z\beta +\tilde{X}\Gamma +E,\;s.t.\;\;1_{p}^{T}A\times \Gamma =0_{q} \end{array}$$

**Figure 1.**
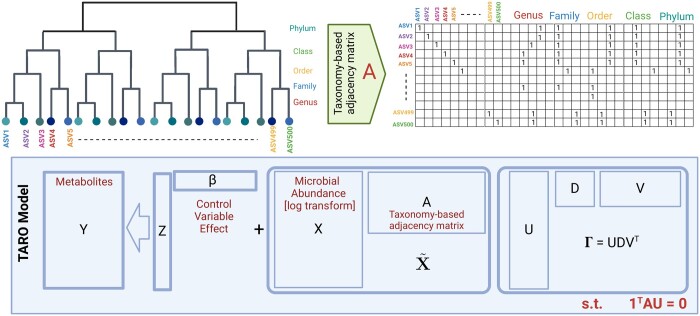
Overview of the TARO model. The taxonomic tree relating the microbiome features (upper left panel) can be encoded as an adjacency matrix **A** (upper right panel). In the matrix **A**, entry *a_ju_* = 1 if feature *j* belongs to the set of leaves descending from node *u* or, for leaf nodes, if *j *=* u*. The TARO factor model (lower panel) relates the metabolite abundances **Y** to the covariates **Z** and aggregated microbiome features $\tilde{X}=XA$, which have a low-rank coefficient matrix Γ.

Since $\Gamma_{(|T|-1)\times q}$ is high dimensional, it is critical to leverage assumptions on its structure to reduce the number of parameters to be estimated. To do so, we build on the framework of reduced-rank regression (Izenman 1975, Chen and Huang 2012). The key idea of reduced-rank regression is to impose a constraint on the rank, or number of linearly independent rows or columns, of Γ, such that r=rank(Γ)<min((|T|−1),q). Following recent advances in factor regression modeling (Mishra *et al.* 2017), we express Γ as a low-rank and sparse coefficient matrix using the components from the singular value decomposition (SVD):

(3) $$\Gamma =UDV^{T}\;\;s.t.\;\;U^{T}U=I,V^{T}V=I,$$

where the left singular vectors are given by $U=[u_{1},\ldots ,u_{r}]$, the right singular vectors are given by $V=[v_{1},\ldots ,v_{r}]$ and the singular values are given by $\text{diag}(D)=[d_{1},\ldots ,d_{r}]$. A schematic overview of the TARO model is shown in Fig. 1.

### 2.2 Sequential estimation of TARO

We now describe our efficient computational procedure for obtaining estimates of the model parameters. With the rank *r* of the coefficient matrix Γ specified, the model parameters can be estimated by solving the optimization problem:

(4) $$\begin{array}{c} \text{arg}{\text{min}}_{\beta ,U,D,V}\|Y-Z\beta +\tilde{X}\Gamma {\|}_{2}^{2}+\rho_{\lambda_{1}}(U)+\rho_{\lambda_{2}}(V),\\ \;\;s.t.\;1_{p}^{T}A\Gamma =0_{q},\Gamma =UDV^{T},U^{T}U=I,V^{T}V=I, \end{array}$$

where $\rho_{\lambda_{1}}(\cdot )$ and $\rho_{\lambda_{2}}(\cdot )$ are sparsity inducing penalties with tuning parameters *λ*_1_ and *λ*_2_, respectively. In high-dimensional settings, sparse estimates of the singular vectors facilitate better model interpretation. With the rank of C unknown and an orthogonality constraint on the singular vectors {U,V}, joint estimation of the parameters is a notoriously intractable problem (Chen 2011, Mishra *et al.* 2017, 2021). However, when orthogonality constraints are dropped, the singular vectors become unidentifiable. As a result, the sparsity pattern in the singular vectors is not unique, which hinders model interpretation. Following the work of Mishra *et al.* (2017), we overcome the challenge by using a sequential approach to estimate the model parameters. Under this approach, we express Γ as the sum of *r* unit-rank matrices:

$$\Gamma =\sum_{i=1}^{r}\Gamma_{i}=\sum_{i=1}^{r}d_{i}u_{i}v_{i}^{T},$$

where $\left\{d_{i},u_{i},v_{i}\right\}$ are SVD components. The estimation procedure then estimates the SVD components $\left\{d_{i},u_{i},v_{i}\right\}$ of Γ in sequential order.

**Step 1**

Extract the first components: With the aim to estimate $\left\{d_{1},u_{1},v_{1},\beta \right\}$, we solve the optimization problem:

$$\begin{array}{c} \hat{\beta },{\hat{d}}_{1},{\hat{u}}_{1},{\hat{v}}_{1}\equiv \text{arg}{\text{min}}_{\beta ,d_{1},u_{1},v_{1}}\|Y-Z\beta +\tilde{X}\Gamma_{1}{\|}_{2}^{2}+\rho_{\lambda }({\tilde{\Gamma }}_{1}◦\Gamma_{1}),\\ s.t.\;\Gamma_{1}{=d}_{1}u_{1}v_{1}^{T},1_{p}^{T}Au_{1}=0,\|u_{1}\|=1,\|v_{1}\|=1, \end{array}$$

where $\rho_{\lambda }({\tilde{\Gamma }}_{1}◦\Gamma_{1})$ is a weighted adaptive elastic-net penalty (Mishra *et al.* 2017) with weights ${\tilde{\Gamma }}_{1}={\left[{\tilde{\gamma }}_{1}^{ij}\right]}_{p\times q}$ inducing sparsity of both the left and right singular vectors $\left\{u_{1},v_{1}\right\}$. Details on the construction of the weights ${\tilde{\Gamma }}_{1}$ and the formulation of the weighted penalty are given in Supplementary Section S1.

**Step k**

Extract the *k*th components: With the aim to estimate $\left\{d_{k},u_{k},v_{k},\beta \right\}$, we solve the optimization problem:

(5) $$\begin{array}{c} \hat{\beta },{\hat{d}}_{k},{\hat{u}}_{k},{\hat{v}}_{k}\equiv \text{arg}{\text{min}}_{\beta ,d_{k},u_{k},v_{k}}\|Y_{k}-Z\beta +\tilde{X}\Gamma_{k}{\|}_{2}^{2}+\rho_{\lambda }({\tilde{\Gamma }}_{k}◦\Gamma_{k}),\\ s.t.\;\Gamma_{k}{=d}_{k}u_{k}v_{k}^{T},1_{p}^{T}Au_{k}=0,\|u_{k}\|=1,\|v_{k}\|=1,\\ U_{1:k-1}^{T}u_{k}=0,V_{1:k-1}^{T}v_{k}=0, \end{array}$$

where $Y_{k}=Y-\tilde{X}\sum_{i=1}^{k-1}{\hat{d}}_{i}{\hat{u}}_{i}{\hat{v}}_{i}^{T}$ is the deflated response matrix and ${\tilde{\Gamma }}_{k}$ is the weight matrix for constructing the sparsity inducing penalty. Motivated by the constraints in the optimization problem (Equation 4), the additional constraints $U_{1:k-1}^{T}u_{k}=0,V_{1:k-1}^{T}v_{k}=0$ are required for imposing orthogonality on the estimate of the singular vectors. Such constraints are necessary in the optimization (Equation 4) for the estimates to be identifiable. However, in the sequential approach one can safely drop the additional constraints and still have an estimate of the singular vectors with a unique sparsity pattern. Hence, to extract the *k*th SVD components, we solve the optimization problem:

$$\begin{array}{c} \hat{\beta },{\hat{d}}_{k},{\hat{u}}_{k},{\hat{v}}_{k}\equiv \text{arg}{\text{min}}_{\beta ,d_{k},u_{k},v_{k}}\|Y_{k}-Z\beta +\tilde{X}\Gamma_{k}{\|}_{2}^{2}+\rho_{\lambda }({\tilde{\Gamma }}_{k}◦\Gamma_{k}),\\ s.t.\;\Gamma_{k}{=d}_{k}u_{k}v_{k}^{T},1_{p}^{T}Au_{k}=0,\|u_{k}\|=1,\|v_{k}\|=1. \end{array}$$

We write the general form of the optimization problem in any *k*th step of the sequential procedure as:

(6) $$\begin{array}{c} \hat{\beta },\hat{d},\hat{u},\hat{v}\equiv \text{arg}{\text{min}}_{\beta ,d,u,v}\|Y_{k}-Z\beta +\tilde{X}\Gamma {\|}_{2}^{2}+\rho_{\lambda }({\tilde{\Gamma }}_{k}◦\Gamma ),\\ s.t.\;\Gamma =duv^{T},1_{p}^{T}Au=0,\|u\|=1,\|v\|=1, \end{array}$$

where $Y_{k}=Y-\tilde{X}\sum_{i=1}^{k-1}{\hat{d}}_{i}{\hat{u}}_{i}{\hat{v}}_{i}^{T}$ is the deflated response matrix. We conveniently represent the unit-rank estimation problem for TARO as $\text{URE-TARO}(d,u,v,\beta ;\tilde{X},Z,Y_{k},A,{\tilde{\Gamma }}_{k})$. We summarize the sequential procedure for parameter estimation in Algorithm 1, with additional details, including a description of tuning parameter selection, provided in Supplementary Section S2.

In applying TARO, users are only required to specify the maximum rank of the coefficient matrix Γ. Utilizing a sequential approach, the computational procedure stops automatically at the *k*th step upon the detection of an estimated singular value ${\hat{d}}_{k}=0$. This approach allows for a systematic determination of the number of latent factors while minimizing computational complexity and ensuring efficiency.

TARO assumes that the errors are uncorrelated and normally distributed; violations of these assumptions will degrade model performance (see Supplementary Section S3.1.2). Deletion diagnostics, which measure the influence of each data point by considering models fitted with versus without the observation, offer a potentially useful approach to characterizing the robustness of the fitted TARO model (Rajaratnam *et al.* 2019). Model diagnostics for reduced-rank regression, including leverage scores, offer another possible approach for identifying influential points (Chen 2016).

Algorithm 1. Tree-Aggregated factor RegressiOn (TARO)

1: Initialization: set *k *=* *1 and set a desired rank r≥1.

2: **repeat**

3:   (1) Set the adaptive weights as ${\tilde{\Gamma }}_{k}={\tilde{d}}_{k},{\tilde{u}}_{k},{\tilde{v}}_{k}$.

4:   (2) Compute the current residual matrix **Y**_*k*_ as in (5).

5:   (3) Perform the $\text{URE-TARO}(d,u,v,\beta ;\tilde{X},Z,Y_{k},A,{\tilde{\Gamma }}_{k})$  analysis via (6) (including the tuning process), and obtain  $\hat{\beta },{\hat{d}}_{k},{\hat{u}}_{k},{\hat{v}}_{k}$.

6:   **if**${\hat{d}}_{k}=0$**then**

7:    Set ${\hat{d}}_{h}=0$ for any k≤h≤r; k←r+1.

8:   **else**

9:    k←k+1.

10:   **end if**

11: **until**k=r+1.

12: **return**${\hat{\Gamma }}_{k},\hat{\beta }$ and $\left({\hat{d}}_{k},{\hat{u}}_{k},{\hat{v}}_{k}\right)$ for all k=1,…,r with ${\hat{d}}_{k}\neq 0$.

### 2.3 Interpretation of the fitted model

The TARO procedure identifies a set of *r* latent factors. Although these factors are learned from patterns of statistical covariation, they can be interpreted as representing biological processes with interplay between the microbiome and metabolome. A natural approach to interpreting the latent factors is to identify the microbiome features and metabolites that participate in each factor. Since TARO imposes sparsity on the loadings vectors, for the *i*th factor, this can be achieved by identifying the nonzero elements of $u_{i}$ and $v_{i}$. Since $u_{i}$ is a vector of length |T|−1, it includes weights for both the leaf and the internal nodes in the tree, corresponding to the observed and aggregated features. It is possible that a leaf node may be selected along with one of its ancestors in the tree; in this case, the ancestor node coefficient can be interpreted as the common effect of related organisms, and the leaf node coefficient represents the unique offset for a specific strain or species. To understand the metabolic patterns represented by each latent factor, coefficients for the selected metabolite features from $v_{i}$ can be used as inputs to metabolite set enrichment analysis, which provides a ranking of metabolic pathways that may be represented (Xia and Wishart 2010). Finally, the scores for each sample on the latent factors may be correlated with clinical covariates to provide context on the clinical relevance of the factors.

## 3 Results

### 3.1 Simulation study

To assess the performance of TARO in comparison to alternative approaches, we carried out a series of simulation studies. The generation of synthetic microbiome profiles is a challenging task due to the complex data structure of microbiome compositional profiles obtained from specimens. To simulate realistic microbial abundances, we relied on SparseDOSSA2 (Ma *et al.* 2021), which utilizes a real data template as a target for the marginal feature distributions. As our template, we relied on the stool profiles from the expanded Human Microbiome Project (Lloyd-Price *et al.* 2017). We then scaled and log-transformed the resulting counts W to obtain our X matrix. We generated a coefficient matrix Γ with true rank *r *=* *3 based on the unit-rank components $\left\{d_{i},u_{i},v_{i}\right\}$ for 1≤i≤r. Here, *d_i_* are the scalars that make up the diagonal of **D**, while **u**_*i*_ and **v**_*i*_ are vectors that will be stacked to obtain **U** and **V**, which each have rank *r*. We therefore refer to these vectors as unit-rank components. We set $d_{1}=4,\;d_{2}=3$, and $d_{3}=2$, and simulated sparse and nearly orthogonal $U=[u_{1},u_{2},u_{3}]$ and $V=[v_{1},v_{2},v_{3}]$ matrices. The final coefficient matrix is generated as $\Gamma =UDV^{T}.$ We utilized a taxonomic tree T obtained from the real data, provided in the TARO R package. We constructed four different settings; in each setting, the true signal is sparse, with only 5% of features affecting the multivariate outcome, but the set of important features differs in its properties. Specifically, we simulate the unit-rank components of the coefficient matrix such that the following feature sets are relevant: (a) features with higher variation, (b) rare features, (c) fine-resolution features (leaf nodes), and (d) aggregated features (internal nodes). Details on the rules used to define these feature sets can be found in Supplementary Table S1. Under our simulation design, features with higher variation will explain a greater proportion of variation in the outcome; from a biological perspective, high variation features may reflect shared environmental exposures or core metabolic processes. Rare features, which may arise from unique exposures or potential pathogens, are more difficult to identify statistically. Fine resolution features reflect differences in function across strains, while aggregated features reflect groups of related features with common function.

We set the sample size *n* to 300. The number of microbial genera *p* varied in the range from 200 to 225 depending on the construction of C. Finally, we generated the response matrix Y using the true model (1) with error term E simulated at a signal-to-noise ratio (SNR) of 0.5, following the definition of the SNR in Mishra *et al.* (2017). We also consider a more challenging set-up where the response matrix Y is generated from the unobserved true abundances, while the observed abundances are provided as input to TARO (Supplementary Section S3.1.3).

To provide insight into the relative performance of TARO, we consider several alternative procedures:

**TRAC**: tree-aggregation of compositional data (Yan and Bien 2021), which is designed for a single outcome.

**CRRR**: linear-constrained reduced-rank regression, a simplified version of TARO without feature selection.

**SeCURE**: sequential co-sparse factor regression (Mishra *et al.* 2017), which is not designed for the microbiome setting.

For an overview of the method properties, see Table 1. Comparing TARO with the marginal approach of TRAC emphasizes the relevance of joint modeling of the multivariate outcome, while the comparison to CRRR highlights the significance of the sparsity-inducing penalty when compared with TARO. Finally, the comparison to SeCURE showcases the importance of imposing linear constraints due to compositionality.

**Table 1.** Summary of models compared, including TARO (tree-aggregated factor regression), TRAC (tree-aggregation of compositional data), CRRR (linear-constrained reduced-rank regression), and SeCURE (sequential co-sparse factor regression).

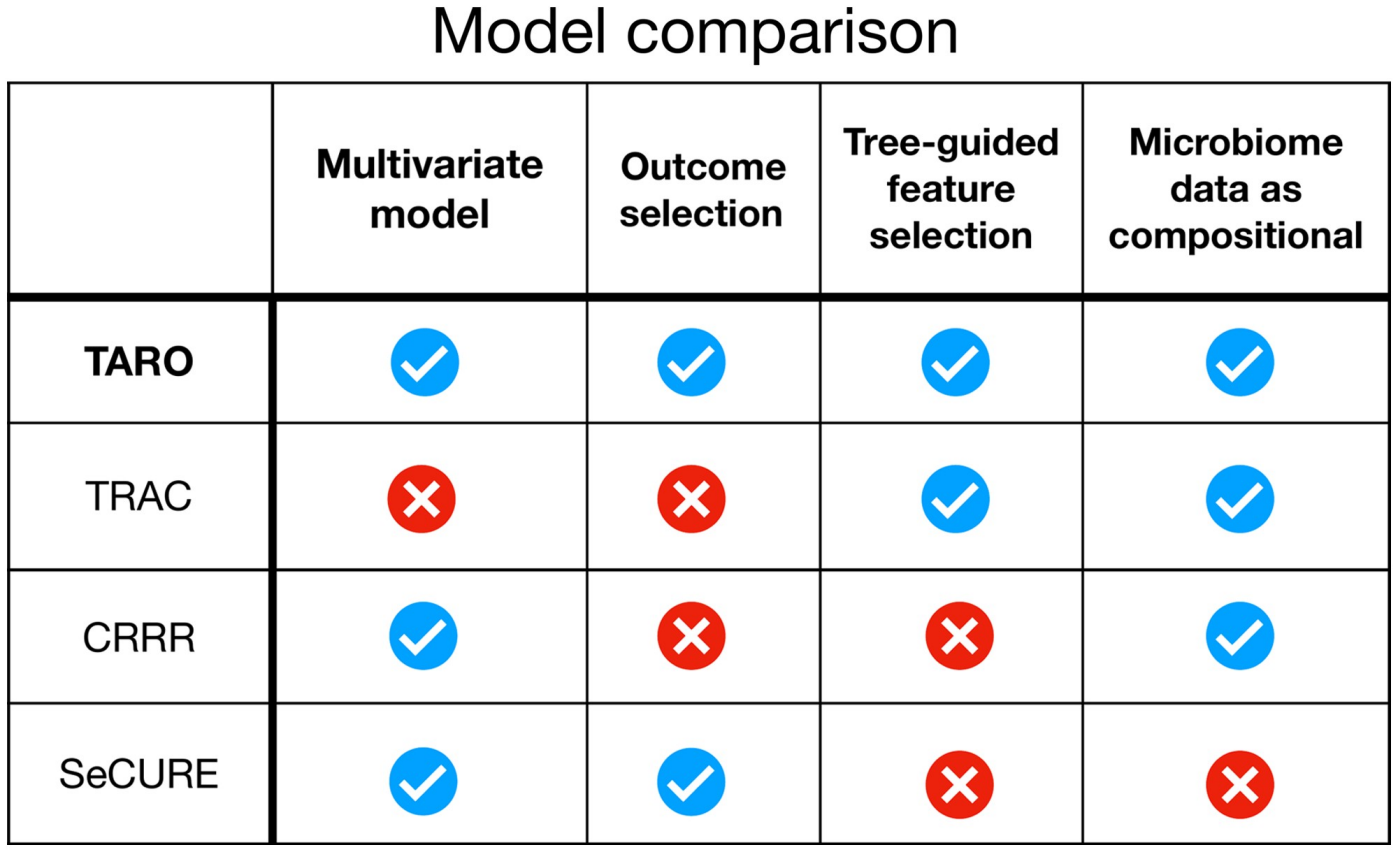

We compare the model results in terms of error in estimating the coefficients $\text{Er}(C)=\left\|\hat{C}-C\right\|$, prediction error $\text{Er}(\text{XC})=\left\|X\hat{C}-XC\right\|$, and feature selection. Performance in feature selection is based on comparing the sparsity pattern of $\left\{{\hat{u}}_{k},{\hat{v}}_{k}\right\}$ to $\left\{u_{k},v_{k}\right\}$ in terms of the false positive rate (FPR) and false negative rate (FNR).

**Figure 2.**
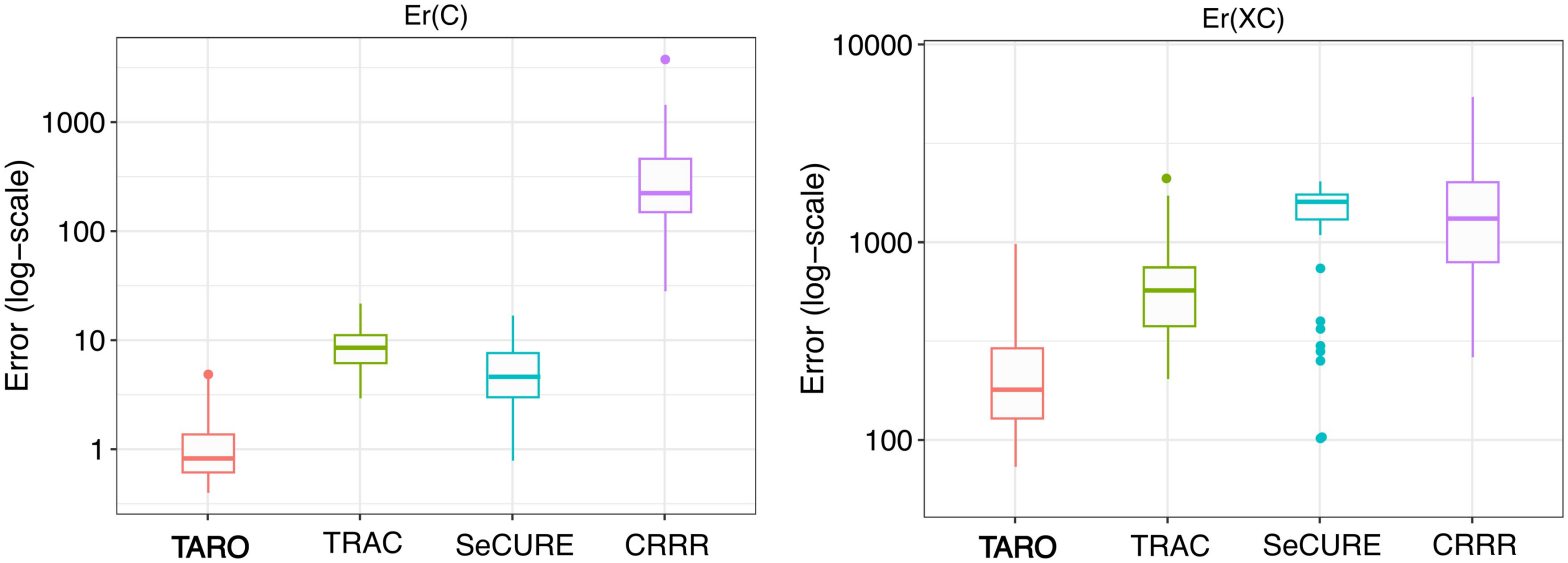
Boxplots of the coefficient estimation error $\text{Er}(C)=\left\|\hat{C}-C\right\|$ and prediction error $\text{Er}(\text{XC})=\left\|X\hat{C}-XC\right\|$ across 50 replicates of simulation setting (a), where higher variation features are relevant to the outcome.

Across 50 replicates of setting (a), where features with higher variation are associated with the multivariate outcomes, TARO achieves consistently lower estimation and prediction error than alternative methods (Fig. 2). TARO also achieves a reasonable balance between the FNR and FPR for feature selection (Table 2). Results from other simulation settings are provided in Supplementary Fig. S1.

**Table 2.** Performance comparison in terms of coefficient estimation error Er(C), prediction error Er(XC), and the false positive rate (FPR) and false negative rate (FNR) for feature selection across 50 replicates of simulation setting (a).

Model	Er(C)	Er(XC)	FNR	FPR
**TARO**	**1.3**	**240**	**0.180**	**0.068**
TRAC	9.1	650	0.031	0.670
CRRR	410.0	1800	0.000	1.00
SeCURE	5.2	1400	0.890	0.010

The performance metrics for TARO are in bold.

Compared to existing approaches for modeling the multivariate outcome with compositional covariates as predictors, TARO demonstrates superior performance in estimation error, prediction error, and sparsity recovery. In high-dimensional settings where the underlying association can be expressed in terms of a low-rank and sparse coefficient matrix, the superior performance of TARO to CRRR and TRAC shows the usefulness of joint modeling of multivariate outcomes and the sparsity-inducing penalty.

### 3.2 Analysis of colorectal cancer data using TARO

There is increasing evidence that the human gut microbiome influences diseases including colorectal cancer and inflammatory bowel disease through the production of metabolites (Lee-Sarwar *et al.* 2020). To provide insight into microbial-metabolite relationships in the gut ecosystem, we applied TARO to analyze metagenomic and metabolomic profiling data collected from participants undergoing colonoscopy as part of a large-scale study in colorectal cancer (Yachida *et al.* 2019). A processed version of this data is provided through the curated gut microbiome-metabolome data resource https://github.com/borenstein-lab/microbiome-metabolome-curated-data/ (Muller *et al.* 2022). The processed data include observations for *n *=* *347 participants on *q *=* *249 metabolites and *p *=* *1456 microbial genera. We defined the adjacency matrix A using the taxonomic tree relating the observed genus-level features.

**Figure 3.**
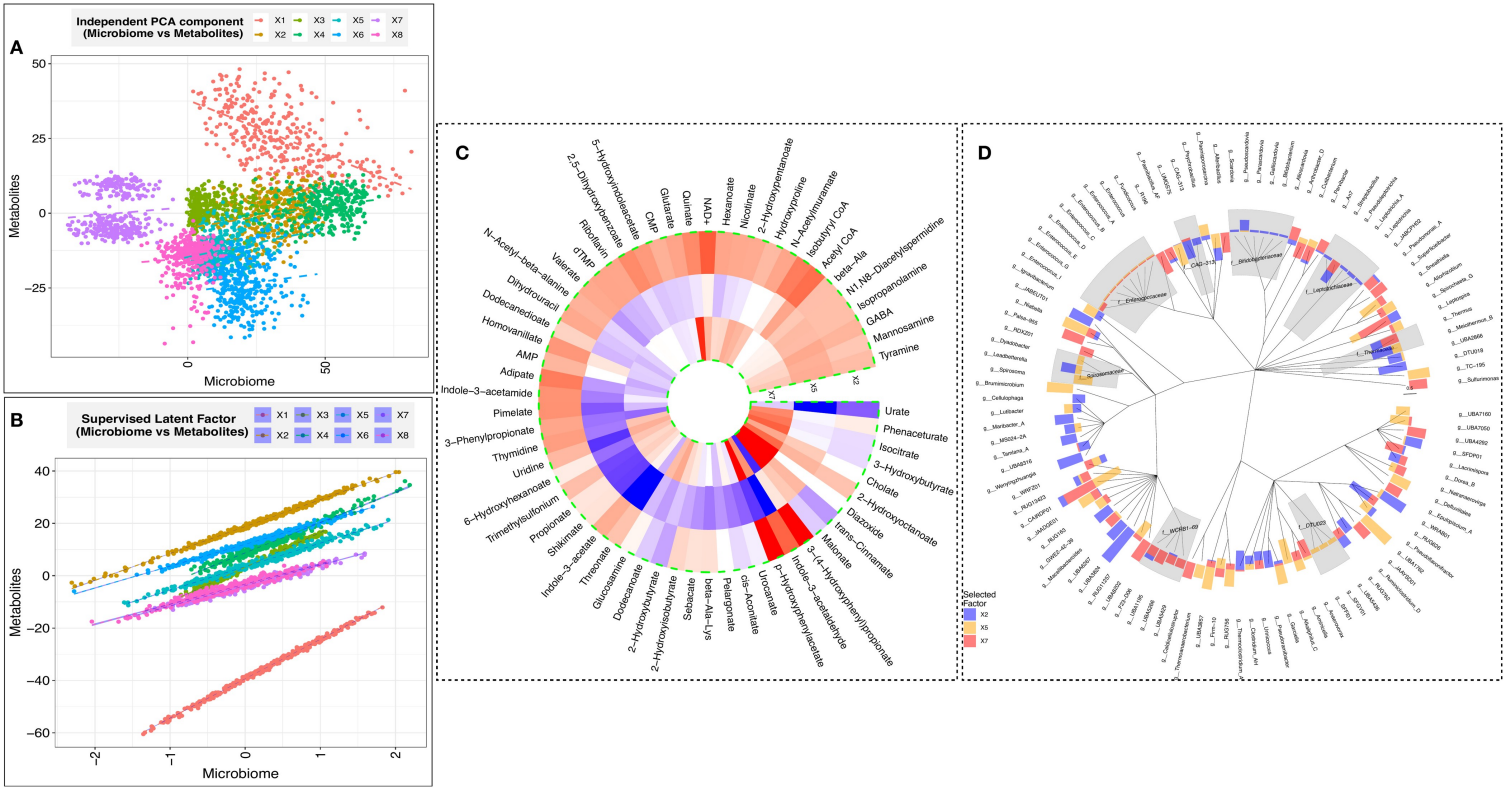
(A) Scores for each sample from PCA conducted independently on each modality, where the colors represent the leading eight components and each point represents a sample. (B) Scores for each sample from TARO model, where the colors represent latent factors. (C) Heatmap of coefficients of selected metabolites for clinically relevant factors (X2, X5, and X7), where red values represent positive loadings and blue values represent negative loadings. (D) Circular barplot of coefficients of selected microbiome features for clinically relevant factors, where the colors correspond to the latent factor (X2, X5, or X7) and the bar heights correspond to the estimated loadings.

To provide new insight into this complex dataset, we applied TARO to characterize the interplay between microbiome profiles and metabolites. As a contrast to TARO, which performs a joint analysis to identify latent factors, we applied sparse principal component analysis (PCA) (Erichson *et al.* 2020) separately on the X and Y matrices. Our goal was to find sets of microbiome features and metabolites that work together to impact phenotypes of interest. We computed the top eight principal components from independent PCA and aligned the components based on their correlation (Fig. 3A). However, the resulting cloud of points suggests that the independently inferred latent components may be capturing distinct activity within each modality, rather than shared processes.

Using TARO, we were able to identify association patterns represented by a low-rank and sparse estimate of the coefficient matrix. Each unit-rank component within the coefficient matrix provides valuable information regarding the subset of metabolites (via the sparse estimate of the loading matrix V) that directly correspond to a subset of microbiome features (via the sparse estimate of U). Upon multiplication of the TARO model with the loading matrix V, it becomes evident that the latent factor XAU showcases a linear relationship with the response factors YV, where the slope is determined by the diagonal elements of matrix **D**. The latent factors identified by TARO capture microbiome-metabolite relationships more efficiently than those from independent PCA, as each latent factor represents a set of microbiome and metabolite features working in concert (Fig. 3B).

Each of the eight latent factors identified by TARO represents distinct sets of microbes and metabolites working together to perform specific tasks in the gut. To identify the latent factors with the greatest clinical relevance, we fit a logistic regression model with the scores on the latent factors as predictors and sample classification (colorectal cancer versus normal tissue) as the response. We adjusted for age, sex, and BMI in the model. Three latent factors (X2, X5, and X7) were significant (*P *<* *.05) in the logistic regression model (Supplementary Fig. S6). TARO selects a sparse set of metabolites (Fig. 3C) and microbiome features (Fig. 3D) that contribute to these clinically relevant latent factors. TARO identifies both both genus-level and also aggregated features as important (Fig. 3D).

We next sought to characterize possible biological processes represented by these latent factors. Based on metabolite set enrichment analysis (Supplementary Fig. S7), we identified propanoate metabolism as a key metabolic pathway represented by X2. Propionate is an abundant short-chain fatty acid in the gut, and altered propionate metabolism has been linked to cancer progression and aggressiveness (Gomes *et al.* 2022). The family Bifidobacteriaceae, which includes the genus *Bifidobacterium*, was identified as an aggregated feature that contributes to X2 (Fig. 3D). Although Bifidobacteriaceae do not directly produce propionate, they contribute to propionate and butyrate production through cross-feeding in the gut (Scott *et al.* 2014). Enrichment analysis revealed that X5 captures functions related to energy metabolism including the citric acid cycle and beta oxidation of very long chain fatty acids. The genus *UBA1762* was identified as a microbial feature contributing to X5; *UBA1762* belongs to the family Ruminococcaceae, which has been previously identified as a taxonomic feature positively associated with response to cancer immunotherapy (Gopalakrishnan *et al.* 2018). Finally, pyrimidine metabolism, which has been closely linked with cancer progression (Wang *et al.* 2021), was identified as a key pathway in X7. Interestingly, several cancer drugs, including the chemotherapeutic agent fluoropyrimidine, act to disrupt pyrimidine metabolism (Spanogiannopoulos *et al.* 2022). The family *WCHB1.69*, which belongs to the order Bacteroidales, is identified as an important microbial feature for X7; Bacteroidales play an important role in shaping response to immmunotherapy (Vétizou *et al.* 2015).

In summary, TARO enables us to identify a small number of latent factors that are relevant to colorectal cancer status and the specific microbiome and metabolite features represented in each factor. TARO enables the formulation of testable hypotheses regarding the interplay between the microbiome and metabolome. The TARO results highlight potential avenues of intervention that can be further explored through pre-clinical studies in mice.

## 4 Conclusion

TARO provides an effective tool for the integration of microbiome and metabolite datasets. Through a specially designed penalization approach, TARO is able to identify specific features from each modality that contribute to a small set of latent factors. Importantly, TARO respects unique aspects of microbiome data including its compositionality and the tree-structured relationships among features. We illustrate the superior performance of TARO in simulation settings and discuss its application to a colorectal cancer dataset.

More broadly, TARO may be applied for the integration of microbiome profiles with high-dimensional data types other than metabolomics. For example, Y could instead represent microbial functional proteins (metaproteomics) or host-associated factors such as immune cell abundances. An interesting possible extension of TARO would be to acknowledge structure among the Y variables, such as pathway membership or network relations, in addition to structure on the X.

## Supplementary Material

btae321_Supplementary_Data

## Data Availability

The metabolite and microbiome profiles analyzed in the case study were originally described in Yachida *et al.* (2019). A processed version of this dataset has been shared through the curated gut microbiome-metabolome data resource (Muller *et al.* 2022) and is available online at https://github.com/borenstein-lab/microbiome-metabolome-curated-data/wiki/.
