# Supplementary material for: TARO: tree-aggregated factor regression for microbiome data integration
Source: Bioinformatics. 2024 May 24;40(6):btae321. doi: 10.1093/bioinformatics/btae321 (PMC11193058; doi:10.1093/bioinformatics/btae321)
Supplement: btae321_Supplementary_Data [file btae321_supplementary_data.pdf]

Supplementary materials for "TARO: tree-aggregated factor regression for microbiome data integration"

Aditya Mishra, Iqbal Mahmud, Philip Lorenzi, Robert Jenq, Jennifer Wargo, Nadim Ajami, and Christine Peterson

Contents

S1 Weighted adaptive-elastic-net penalty 1

S2 URE-TARO procedure 2
- S2.1 Constrained adaptive elastic-net solution . . . 2
- S2.2 Constrained reduced-rank regression . . . 4
- S2.3 Tuning parameter selection . . . 5

S3 Supplementary figures 5
- S3.1 Simulation results . . . 5
  - S3.1.1 Robustness to choice of pseudocount . . . 5
  - S3.1.2 Robustness to model misspecification . . . 7
  - S3.1.3 Evaluation of TARO with unobserved true abundance . . . 8
- S3.2 Application results . . . 9

S1. Weighted adaptive-elastic-net penalty

We use the adaptive elastic net penalty [Zou and Hastie, 2005, Zou and Zhang, 2009],

$$\rho(\mathbf{C};\lambda) = \rho(\mathbf{C};\mathbf{W}_1,\lambda,\alpha) = \alpha\lambda\|\mathbf{W}_1 \circ \mathbf{C}\|_1 + (1-\alpha)\lambda\|\mathbf{C}\|_F^2$$
$$= \alpha\lambda\sum_{i=1}^{p}\sum_{j=1}^{q} w_{ij1}|c_{ij}| + (1-\alpha)\lambda\sum_{i=1}^{p}\sum_{j=1}^{q} c_{ij}^2. \tag{1}$$

Here $\|\cdot\|_1$ denotes the ℓ_1 norm, the operator "$\circ$" stands for the Hadamard product, $\mathbf{W}_1 = [w_{ij1}]_{p\times q}$ is a pre-specified weighting matrix, λ is a tuning parameter controlling the overall amount of regularization, and $\alpha \in (0,1)$ controls the relative weights between the two penalty terms. We set $\mathbf{W}_1 = |\widetilde{\mathbf{C}}_1|^{-\gamma}$ such that $w_{ij1} = w_1^{(d)} w_{i1}^{(u)} w_{j1}^{(v)}$, with

$$w_1^{(d)} = |\widetilde{d}_1|^{-\gamma}, \mathbf{w}_1^{(u)} = [w_{11}^{(u)},...,w_{p1}^{(u)}]^{\mathrm{T}} = |\widetilde{\mathbf{u}}_1|^{-\gamma}, \mathbf{w}_1^{(v)} = [w_{11}^{(v)},...,w_{q1}^{(v)}]^{\mathrm{T}} = |\widetilde{\mathbf{v}}_1|^{-\gamma}, \tag{2}$$

where $\widetilde{\mathbf{C}}_1 = \widetilde{d}_1\widetilde{\mathbf{u}}_1\widetilde{\mathbf{v}}_1^{\mathrm{T}}$ is the first set of unit-rank RRR estimators and γ is a non-negative constant with $|\cdot|^{-\gamma}$ componentwisely defined.

S2. URE-TARO procedure

Here, we provide additional detail on the unit rank estimation procedure for TARO described in Section 2.2 of the main manuscript. In our previous work on sparse factor regression Mishra et al. [2021], we imposed sparsity directly on $\mathbf{C}$. To enable appropriate aggregation of the microbiome features in the factor regression framework, we instead propose to impose sparsity on $\boldsymbol{\Gamma}$.

$$\begin{aligned} \widehat{\boldsymbol{\beta}}, \widehat{d}, \widehat{\mathbf{u}}, \widehat{\mathbf{v}} \;&\equiv\; \underset{\boldsymbol{\beta}, d, \mathbf{u}, \mathbf{v}}{\arg\min}\; \|\mathbf{Y} - \mathbf{Z}\boldsymbol{\beta} + \widetilde{\mathbf{X}}\boldsymbol{\Gamma}\|_2^2 + \rho_\lambda(\widetilde{\boldsymbol{\Gamma}} \circ \boldsymbol{\Gamma}), \\ \text{s.t.}\quad & \boldsymbol{\Gamma} = d\mathbf{u}\mathbf{v}^{\mathrm{T}}, \mathbf{1}_p^T \mathbf{A}\mathbf{u} = 0, \|\mathbf{u}\| = 1, \|\mathbf{v}\| = 1, \end{aligned} \tag{3}$$

In terms of the parameters $\{\boldsymbol{\beta}, d, \mathbf{u}, \mathbf{v}\}$, the optimization problems of URE-TARO is a multi-convex problem. We define the weighted penalty as:

$$\rho_\lambda(\widetilde{\boldsymbol{\Gamma}}_1 \circ \boldsymbol{\Gamma}_1) = \alpha\lambda \sum_{i,j} |\tilde{\gamma}_1^{ij} d_1 u_{1i} v_{1j}| + (1-\alpha)\|d_1 \mathbf{u}_1 \mathbf{v}_1^{\mathrm{T}}\|_F^2, \tag{4}$$

where λ is the tuning parameter, α provides a relative weights of ℓ_1 and ℓ_2 penalty. We estimate the model parameters using an iterative procedure that cycles between $\mathbf{u}$-step, $\mathbf{v}$-step and $\boldsymbol{\beta}$-step until convergence.

u-step: For the fixed $\mathbf{v}$ and $\boldsymbol{\beta}$, we jointly update $(d, \mathbf{u})$ satisfying $\|\mathbf{v}\| = 1$. Let us define $\check{\mathbf{u}} = d\mathbf{u}$. In terms of $\check{\mathbf{u}}$, the optimization problem (3) is equivalent to solving (constrained adaptive elastic-net problem)

$$\min_{\check{\mathbf{u}}} \left\{ \frac{1}{2}\|\mathbf{y} - \mathbf{X}^{(u)}\check{\mathbf{u}}\|_2^2 + \lambda_1^{(u)} \sum_{i=1}^{p} w_i |\check{u}_i| + \lambda_2^{(u)} \sum_{i=1}^{p} \check{u}_i^2, \text{ s.t. } \mathbf{1}_p^T \mathbf{A}\check{\mathbf{u}} = 0 \right\}, \tag{5}$$

where $\mathbf{y} = \mathrm{vec}(\mathbf{Y} - \mathbf{Z}\boldsymbol{\beta})$, $\mathbf{X}^{(u)} = \mathbf{v} \otimes \mathbf{X}$, $\lambda_1^{(u)} = \alpha\lambda w^{(d)}(\sum_{j=1}^{q} w_j^{(v)}|v_j|)$, and $\lambda_2^{(u)} = (1-\alpha)\lambda \sum_{j=1}^{q} v_j^2$. Here $\mathrm{vec}(\cdot)$ is the vectorization operator, and $\otimes$ denotes the Kronecker product. We recover the singular value estimate as $\hat{d} = \|\check{\mathbf{u}}\|$ and singular vector estimate as $\hat{\mathbf{u}} = \check{\mathbf{u}}/\hat{d}$.

v-step: For fixed $\mathbf{u}$ and $\boldsymbol{\beta}$, we minimize the objective function in terms of the block variable $(d, \mathbf{v})$ such that $\|\mathbf{u}\| = 1$. Let us define $\check{\mathbf{v}} = d\mathbf{v}$. In terms of $\check{\mathbf{v}}$, the optimization problem (3) is equivalent to solving (an adaptive elastic-net problem)

$$\min_{\check{\mathbf{v}}} \left\{ \|\mathbf{y} - \mathbf{X}^{(v)}\check{\mathbf{v}}\|_2^2 + \lambda_1^{(v)} \sum_{j=1}^{q} w_j |\check{v}_j| + \lambda_2^{(v)} \sum_{j=1}^{q} \check{v}_j^2 \right\}, \tag{6}$$

where $\mathbf{X}^{(v)} = \mathbf{I}_q \otimes (\mathbf{X}\mathbf{u})$, $\lambda_1^{(v)} = \alpha\lambda w^{(d)}(\sum_{i=1}^{p} w_i^{(u)}|u_i|)$, and $\lambda_2^{(v)} = (1-\alpha)\lambda \sum_{i=1}^{p} u_i^2$. We recover the singular value estimate as $\hat{d} = \|\check{\mathbf{u}}\|$ and singular vector estimate as $\hat{\mathbf{u}} = \check{\mathbf{u}}/\hat{d}$.

$\boldsymbol{\beta}$-step: For fixed $\{d, \mathbf{u}, \mathbf{v}\}$, unique solution minimizing the objective function is given by

$$\boldsymbol{\beta} = (\mathbf{Z}^{\mathrm{T}}\mathbf{Z})^{-1}\mathbf{Z}^{\mathrm{T}}(\mathbf{Y} + \widetilde{\mathbf{X}}\boldsymbol{\Gamma})$$

S2.1 Constrained adaptive elastic-net solution

Consider a genetic form of the linear constrained adaptive elastic net regression,

$$\min_{\boldsymbol{\beta} \in \mathbb{R}^p} \left\{ J(\boldsymbol{\beta}) \equiv \frac{1}{2}\|\mathbf{y} - \mathbf{X}\boldsymbol{\beta}\|_2^2 + \lambda_1 \sum_{j=1}^{p} w_j |\beta_j| + \frac{\lambda_2}{2} \sum_{j=1}^{p} \beta_j^2 \right\}, \quad \text{s.t. } \mathbf{A}\boldsymbol{\beta} = \mathbf{b}, \tag{7}$$

where $\mathbf{y} \in \mathbb{R}^n$, $\mathbf{X} \in \mathbb{R}^{n \times p}$, $\mathbf{A} = (\mathbf{a}_1, \ldots, \mathbf{a}_p) \in \mathbb{R}^{h \times p}$, $\mathbf{b} \in \mathbb{R}^{h \times 1}$, $\mathbf{w} = [w_1, ..., w_p]^{\mathrm{T}}$ are some predetermined weights, and λ_1 and λ_2 are tuning parameters.

This is an equality-constrained convex optimization problem. The augmented Lagrangian function is

$$L_\mu(\boldsymbol{\beta}, \mathbf{c}) = J(\boldsymbol{\beta}) + \mathbf{c}^{\mathrm{T}}(\mathbf{A}\boldsymbol{\beta} - \mathbf{b}) + \frac{\mu}{2}\|\mathbf{A}\boldsymbol{\beta} - \mathbf{b}\|_2^2,$$

where $\mathbf{c}$ is the Lagrange multiplier, and $\mu > 0$ is a penalty parameter. The iterative steps to solve the problem,

$$\left.\begin{aligned} \boldsymbol{\beta}^{(s+1)} &= \arg\min_{\boldsymbol{\beta}} L_\mu(\boldsymbol{\beta}, \mathbf{c}^{(s)}) \\ &= \arg\min_{\boldsymbol{\beta}} \{J(\boldsymbol{\beta}) + \frac{\mu}{2}\|\mathbf{A}\boldsymbol{\beta} - \mathbf{b} - \frac{\mathbf{c}^{(s)}}{\mu}\|_2^2\}, \\ \mathbf{c}^{(s+1)} &= \mathbf{c}^{(s)} - (\mathbf{A}\boldsymbol{\beta}^{(s+1)} - \mathbf{b})\mu. \end{aligned}\right\} \tag{8}$$

The method converges under very general conditions. As the iteration proceeds, the residual $\mathbf{A}\boldsymbol{\beta}^{s+1} - b$ converges to zero, yielding optimality.

Following (8), the key is to minimize

$$\boldsymbol{\beta}^{(s+1)} = \min_{\boldsymbol{\beta}} \left\{ \frac{1}{2}\|\mathbf{y} - \mathbf{X}\boldsymbol{\beta}\|_2^2 + \lambda_1 \sum_{j=1}^{p} w_j|\beta_j| + \frac{\lambda_2}{2}\|\boldsymbol{\beta}\|_2^2 + \frac{\mu^{(s)}}{2}\|\mathbf{A}\boldsymbol{\beta} - \mathbf{b} - \frac{\mathbf{c}^{(s)}}{\mu^{(s)}}\|_2^2 \right\}. \tag{9}$$

Here the penalty parameter $\mu^{(s)}$ can be updated along the interactions; let $\mu \to \infty$ or increase with small increments can in general improve the speed of convergence [Goldstein and Osher, 2009]. The above problem can be efficiently minimized by a coordinate descent algorithm. Suppose all the β_ks are fixed except β_j, and denote $\mathbf{r}_j = \mathbf{y} - \sum_{k \neq j} \mathbf{x}_k\beta_k$. The objective function with respect to β_j becomes

$$\frac{1}{2}\|\mathbf{r}_j - \mathbf{x}_j\beta_j\|_2^2 + \lambda_1 w_j|\beta_j| + \frac{\mu^{(s)}}{2}\|\mathbf{a}_j\beta_j + \sum_{k \neq j}\mathbf{a}_k\beta_k - \mathbf{b} - \frac{\mathbf{c}^{(s)}}{\mu^{(s)}}\|_2^2 + \frac{\lambda_2}{2}\beta_j^2 + \text{const.}$$

Then it can be easily verified that

$$\widehat{\beta}_j = \frac{\mathcal{S}\left((\mathbf{y} - \sum_{i \neq j}\beta_i\mathbf{x}_i)^{\mathrm{T}}\mathbf{x}_j + \mu^{(s)}\{(\frac{\mathbf{c}^{(s)}}{\mu^{(s)}} + \mathbf{b})^{\mathrm{T}}\mathbf{a}_j - \sum_{i \neq j}\beta_i\mathbf{a}_i^{\mathrm{T}}\mathbf{a}_j\}, \lambda w_j\right)}{\lambda_2 + \mathbf{x}_j^{\mathrm{T}}\mathbf{x}_j + \mu^{(s)}\mathbf{a}_j^{\mathrm{T}}\mathbf{a}_j}, \tag{10}$$

where $\mathcal{S}(m, \lambda) = \text{sign}(m)(|m| - \lambda)_+$ is the soft-thresholding operator. (9) can then be solved by iteratively updating each β_j, $j = 1, \ldots, p$, by (10) until convergence. Our proposed algorithm is presented in Algorithm 1.

Algorithm 1 Bregman Coordinate Descent Algorithm (BCDA)

Initialization: $s = 0$, $\boldsymbol{\beta}^{(0)} \in \mathbb{R}^p$, $\mathbf{c}^{(0)} = \mathbf{0}$, $\mu^{(0)} = 1$, and $\rho \geqslant 1$.

repeat

(1) Use coordinate descent to obtain $\boldsymbol{\beta}^{(s+1)}$ by iteratively updating β_js using (10) until convergence.

(2) $\mathbf{c}^{(s+1)} = \mathbf{c}^{(s)} - (\mathbf{A}\boldsymbol{\beta}^{(s+1)} - \mathbf{b})\mu^{(s)}$.

(3) $\mu^{(s+1)} = \mu^{(s)}\rho$.

$s \leftarrow s + 1$.

until convergence, i.e., $\|\boldsymbol{\beta}^{(s+1)} - \boldsymbol{\beta}^{(s)}\|/\|\boldsymbol{\beta}^{(s)}\| < \epsilon$.

return $\hat{\boldsymbol{\beta}}(\lambda_1, \lambda_2)$.

S2.2 Constrained reduced-rank regression

We consider a general form of the linear-constrained reduced rank regression as

$$\min_{\boldsymbol{\Gamma} \in \mathbb{R}^{p\times q}} \left\{ J(\boldsymbol{\Gamma}) \equiv \frac{1}{2}\|\mathbf{Y} - \mathbf{X}\boldsymbol{\Gamma}\|_F^2 \right\}, \quad \text{s.t.} \quad \text{rank}(\boldsymbol{\Gamma}) = 1,\ \mathbf{A}\boldsymbol{\Gamma} = \mathbf{B}, \tag{11}$$

where $\mathbf{Y} \in \mathbb{R}^{n\times q}$, $\mathbf{X} \in \mathbb{R}^{n\times p}$, $\mathbf{A} = (\mathbf{a}_1, \ldots, \mathbf{a}_p) \in \mathbb{R}^{h\times p}$ and $\mathbf{B} \in \mathbb{R}^{h\times q}$. To solve the optimization problem, we write the augmented Lagrangian function as

$$L_\mu(\boldsymbol{\Gamma}, \boldsymbol{\delta}) = J(\boldsymbol{\Gamma}) + \text{tr}(\boldsymbol{\delta}^{\mathrm{T}}(\mathbf{A}\boldsymbol{\Gamma} - \mathbf{B})) + \frac{\mu}{2}\|\mathbf{A}\boldsymbol{\Gamma} - \mathbf{B}\|_F^2,$$

where $\boldsymbol{\delta} \in \mathbb{R}^{h\times p}$ is the Lagrange multiplier, and $\mu > 0$ is a penalty parameter. The iterative steps to solve the problem,

$$\left.\begin{aligned} \boldsymbol{\Gamma}^{(s+1)} &= \arg\min_{\boldsymbol{\Gamma}} L_\mu(\boldsymbol{\Gamma}, \boldsymbol{\delta}^{(s)}) \quad \text{s.t.} \quad \text{rank}(\boldsymbol{\Gamma}) = 1, \\ &= \arg\min_{\boldsymbol{\Gamma}}\ J(\boldsymbol{\Gamma}) + \frac{\mu}{2}\|\mathbf{A}\boldsymbol{\Gamma} - \mathbf{B} - \frac{\boldsymbol{\delta}^{(s)}}{\mu}\|_2^2 \quad \text{s.t.} \quad \text{rank}(\boldsymbol{\Gamma}) = 1, \\ \boldsymbol{\delta}^{(s+1)} &= \boldsymbol{\delta}^{(s)} - (\mathbf{A}\boldsymbol{\Gamma}^{(s+1)} - \mathbf{B})\mu. \end{aligned}\right\} \tag{12}$$

The method converges under very general conditions. As the iteration proceeds, the residual $\|\mathbf{A}\boldsymbol{\Gamma}^{s+1} - \mathbf{B}\|$ converges to zero, yielding optimality. We simplify the rank constrained $L_\mu(\boldsymbol{\Gamma}, \boldsymbol{\delta}^{(s)})$ as

$$\begin{aligned} L_\mu(\boldsymbol{\Gamma}, \boldsymbol{\delta}^{(s)}) &= \frac{1}{2}\|\mathbf{Y} - \mathbf{X}\boldsymbol{\Gamma}\|_F^2 + \frac{\mu}{2}\|\mathbf{A}\boldsymbol{\Gamma} - \mathbf{B} - \frac{\boldsymbol{\delta}^{(s)}}{\mu}\|_F^2 \\ &= \frac{1}{2}\|\widetilde{\mathbf{Y}} - \widetilde{\mathbf{X}}\boldsymbol{\Gamma}\|_F^2, \end{aligned}$$

where $\widetilde{\mathbf{Y}} = [\mathbf{Y}^{\mathrm{T}}\ \sqrt{\mu}\mathbf{B}^{\mathrm{T}} + \frac{\boldsymbol{\delta}^{(s)\mathrm{T}}}{\sqrt{\mu}}]^{\mathrm{T}}$ and $\widetilde{\mathbf{X}} = [\mathbf{X}^{\mathrm{T}}\ \mathbf{A}^{\mathrm{T}}]^{\mathrm{T}}$. An optimal solution of

$$\arg\min_{\boldsymbol{\Gamma}} \frac{1}{2}\|\widetilde{\mathbf{Y}} - \widetilde{\mathbf{X}}\boldsymbol{\Gamma}\|_F^2 \quad \text{s.t.} \quad \text{rank}(\boldsymbol{\Gamma}) = 1,$$

is given by $\widehat{\boldsymbol{\Gamma}} = (\widetilde{\mathbf{X}}^{\mathrm{T}}\widetilde{\mathbf{X}})^{-1}\widetilde{\mathbf{X}}^{\mathrm{T}}\widetilde{\mathbf{Y}}\tilde{\mathbf{v}}\tilde{\mathbf{v}}^{\mathrm{T}}$ where $\tilde{\mathbf{v}}$ is the largest eigen-vector of $\widetilde{\mathbf{Y}}^{\mathrm{T}}\widetilde{\mathbf{X}}(\widetilde{\mathbf{X}}^{\mathrm{T}}\widetilde{\mathbf{X}})^{-1}\widetilde{\mathbf{X}}^{\mathrm{T}}\widetilde{\mathbf{Y}}$. Our proposed algorithm is presented in Algorithm 2.

Algorithm 2 Linear Constrained Reduced Rank Regression

Initialization: $s = 0$, $\boldsymbol{\Gamma}^{(0)} \in \mathbb{R}^{p\times q}$, $\boldsymbol{\delta}^{(0)} = \mathbf{0}$, $\mu^{(0)} = 1$, and $\rho \geqslant 1$.
repeat
(1) $\boldsymbol{\Gamma}^{(s+1)} = (\widetilde{\mathbf{X}}^{\mathrm{T}}\widetilde{\mathbf{X}})^{-1}\widetilde{\mathbf{X}}^{\mathrm{T}}\widetilde{\mathbf{Y}}\tilde{\mathbf{v}}\tilde{\mathbf{v}}^{\mathrm{T}}$ where $\tilde{\mathbf{v}}$ is the largest eigen-vector of $\widetilde{\mathbf{Y}}^{\mathrm{T}}\widetilde{\mathbf{X}}(\widetilde{\mathbf{X}}^{\mathrm{T}}\widetilde{\mathbf{X}})^{-1}\widetilde{\mathbf{X}}^{\mathrm{T}}\widetilde{\mathbf{Y}}$ such that $\widetilde{\mathbf{Y}} = [\mathbf{Y}^{\mathrm{T}}\ \sqrt{\mu}\mathbf{B}^{\mathrm{T}} + \frac{\boldsymbol{\delta}^{(s)\mathrm{T}}}{\sqrt{\mu}}]^{\mathrm{T}}$ and $\widetilde{\mathbf{X}} = [\mathbf{X}^{\mathrm{T}}\ \mathbf{A}^{\mathrm{T}}]^{\mathrm{T}}$.
(2) $\boldsymbol{\delta}^{(s+1)} = \boldsymbol{\delta}^{(s)} - [\mathbf{A}\boldsymbol{\Gamma}^{(s+1)} - \mathbf{B}]\mu$.
(3) $\mu^{(s+1)} = \mu^{(s)}\rho$.
$s \leftarrow s + 1$.
until convergence, i.e., $\|\boldsymbol{\Gamma}^{(s+1)} - \boldsymbol{\Gamma}^{(s)}\|/\|\boldsymbol{\Gamma}^{(s)}\| < \epsilon$.
return $\widetilde{\boldsymbol{\Gamma}}$.

S2.3 Tuning parameter selection

We have outlined the sequential procedure in Algorithm 1 of the main manuscript. Within each step of this sequential process, we solve a unit-rank estimation (URE-TARO) problem, as described above. With only one tuning parameter, λ, we generate a solution path for various λ values within the range of λ_{max} to λ_{min} (a chosen multiple of λ_{max}), depicted in the first two subplots of Figure S1. Subsequently, we employ k-fold cross-validation (with k=5 recommended) to select the λ value associated with the lowest error, as demonstrated in the rightmost plot of Figure S1.

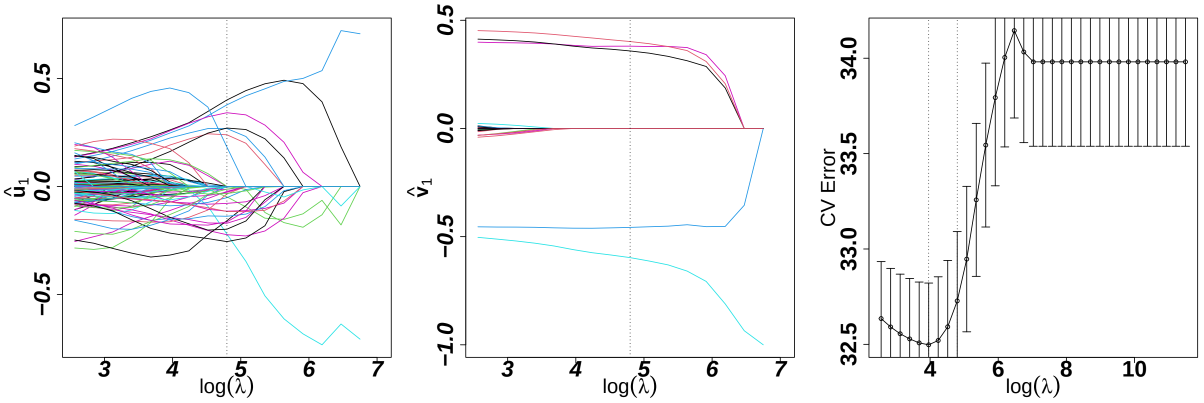

Figure S1: Tuning parameter selection using k-fold cross-validation in TARO. The left subplot represents solution paths for $\hat{\mathbf{u}}_1$, the center subplot represents solution paths for $\hat{\mathbf{v}}_1$, and the right subplot represents the cross validation error for increasing values of λ.

S3. Supplementary figures

S3.1 Simulation results

Here, we provide additional figures summarizing the simulation results for the settings described in Section 3.1 of the main manuscript. Specifically, we considered simulation settings where the unit-rank components of the coefficient matrix are constructed such that the following feature sets are relevant: a) features with higher variation, b) rare features, c) fine-resolution features (leaf nodes), and d) aggregated features (internal nodes). We have provided more details about the four simulation settings in Table S1. The results for setting a) are given in Figure 2 of the main manuscript. The results for the remaining scenarios are provided in Figure S2. TARO consistently outperforms the alternative methods considered in terms of accuracy in the estimation of the true coefficient matrix, prediction accuracy, and recovery of the true features.

S3.1.1 Robustness to choice of pseudocount

By default, TARO assumes a pseudocount of 1. To assess the robustness of TARO to the choice of pseudocount value, we have compared the default setting of TARO with pseudocount values of 0.5, 0.25, and 2 (see Figure S3 for performance comparison). We found that in all four settings, the pseudocount of 2 significantly increases the error estimate Er(C).

In Setting c), we observe an increase in the error estimate with pseudocounts of 0.25 or 0.5 compared to the default pseudocount of 1. Setting c) aims to showcase the model's efficacy in a scenario where the underlying relationship between the multivariate response and predictors is expressed solely in terms of leaf nodes in the taxonomic tree.

Setting 2

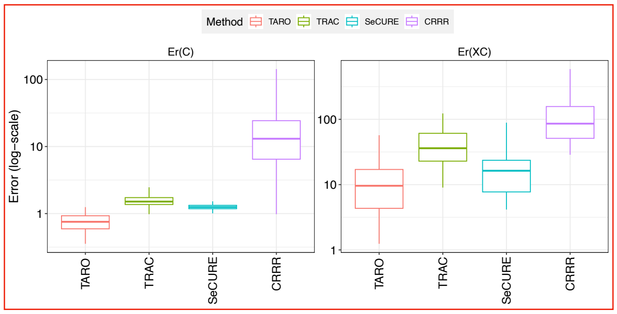

Model	Er(C)	Er(XC)	FNR	FPR
**Full data**				
**TARO**	**0.78**	**13**	**0.26**	**0.061**
TRAC	1.60	45	0.40	0.720
CRRR	21.00	130	0.00	1.000
SeCURE	1.30	20	0.58	0.100

Setting 3

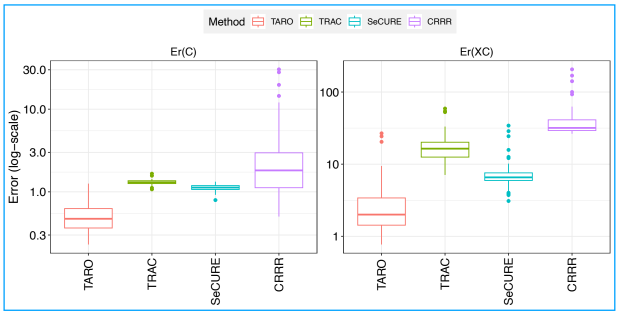

Model	Er(C)	Er(XC)	FNR	FPR
**Full data**				
**TARO**	**0.53**	**3.8**	**0.14**	**0.048**
TRAC	1.30	19.0	0.48	0.710
CRRR	4.20	45.0	0.00	1.000
SeCURE	1.10	8.2	0.54	0.170

Setting 4

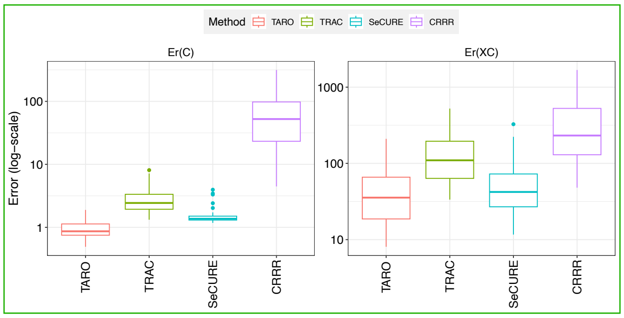

Model	Er(C)	Er(XC)	FNR	FPR
**Full data**				
**TARO**	**0.97**	**46**	**0.37**	**0.11**
TRAC	2.90	140	0.38	0.72
CRRR	74.00	350	0.00	1.00
SeCURE	1.50	61	0.74	0.11

Figure S2: Model performance comparison in various simulated settings in terms of estimation accuracy, prediction, and sparsity recovery.

Table S1: Detail on the simulation settings. Further details regarding the implementation can be obtained from the `taro_sim` function within the R package `taro`.

	**Description**
Setting 1	In this setting, a greater number of leaf taxa in the taxonomic tree affect the outcome. We randomly select 10% of taxa, including both internal nodes and leaves, favoring those with *higher variability* across samples. Specifically, we include one taxon with four leaf nodes as children, one taxon with three leaf nodes as children, and three taxa with two leaf nodes as children. The remaining relevant features come mainly from leaf nodes in the taxonomic tree.
Setting 2	In this setting, a greater number of leaf taxa in the taxonomic tree affect the outcome. We randomly select 10% of taxa, including both nodes and leaves, favoring those with *lower variability* across samples. Specifically, we include one taxon with four leaf nodes as children, one taxon with three leaf nodes as children, and three taxa with two leaf nodes as children. The remaining relevant features come mainly from leaf nodes in the taxonomic tree.
Setting 3	In this setting, only leaf nodes in the taxonomic tree affect the outcome. We randomly select 10% of taxa, favoring those with *lower variability* across samples.
Setting 4	In this setting, a greater number of higher taxa in the taxonomic tree affect the outcome. We randomly select 10% of taxa, including both internal nodes and leaves, favoring those with *lower variability* across samples. Specifically, we include four taxa with four leaf nodes as children, two taxa with three leaf nodes as children, and ten taxa with two leaf nodes as children are deemed relevant. The remaining relevant features come from leaf nodes in the taxonomic tree.

In summary, our analysis indicates that the choice of a pseudocount of 1 is optimal for multivariate analysis using TARO.

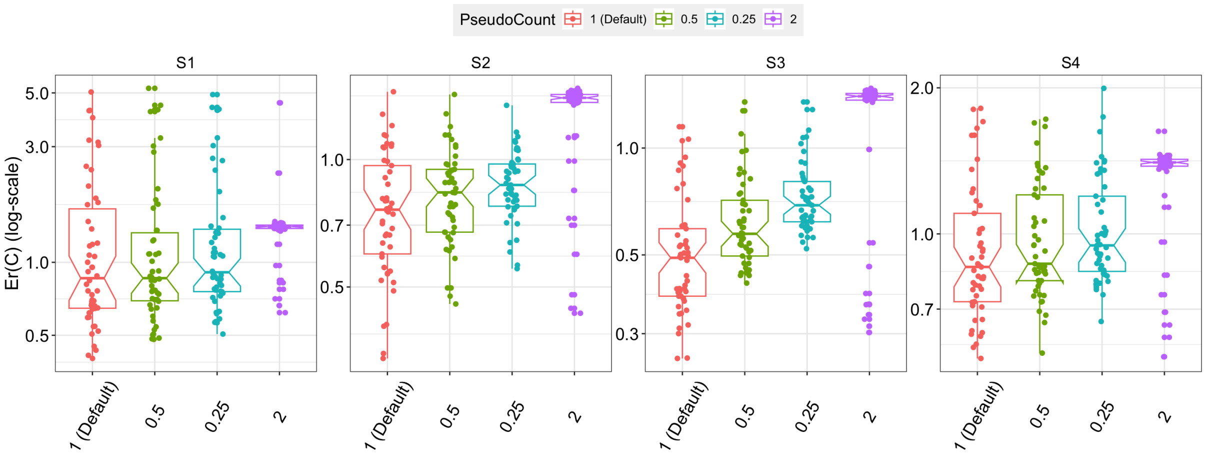

Figure S3: Simulation study: TARO estimation error evaluated for different choices of the pseudocount: 1 (default setting), 0.5, 0,25, and 2.

S3.1.2 Robustness to model misspecification

We have conducted a comprehensive series of simulations where we deliberately introduce model misspecifications. This involved systematically varying both the error structure (including different levels of correlation in the errors among the multivariate responses) and the error distribution (considering heavy-tailed symmetric distributions such as the Laplace, Cauchy, and Student's t distributions). This analysis

aimed to provide insights into our method's ability to maintain accuracy and reliability in real-world scenarios where the underlying assumptions may not hold. We summarize the results in Figure S4.

The performance of TARO deteriorates when there is a high correlation among the multivariate responses or when the errors arise from heavy-tailed distributions. This is because TARO assumes that the errors are independent and normally distributed. The alternative methods considered in the main manuscript make similar assumptions, so this challenge is not unique to TARO.

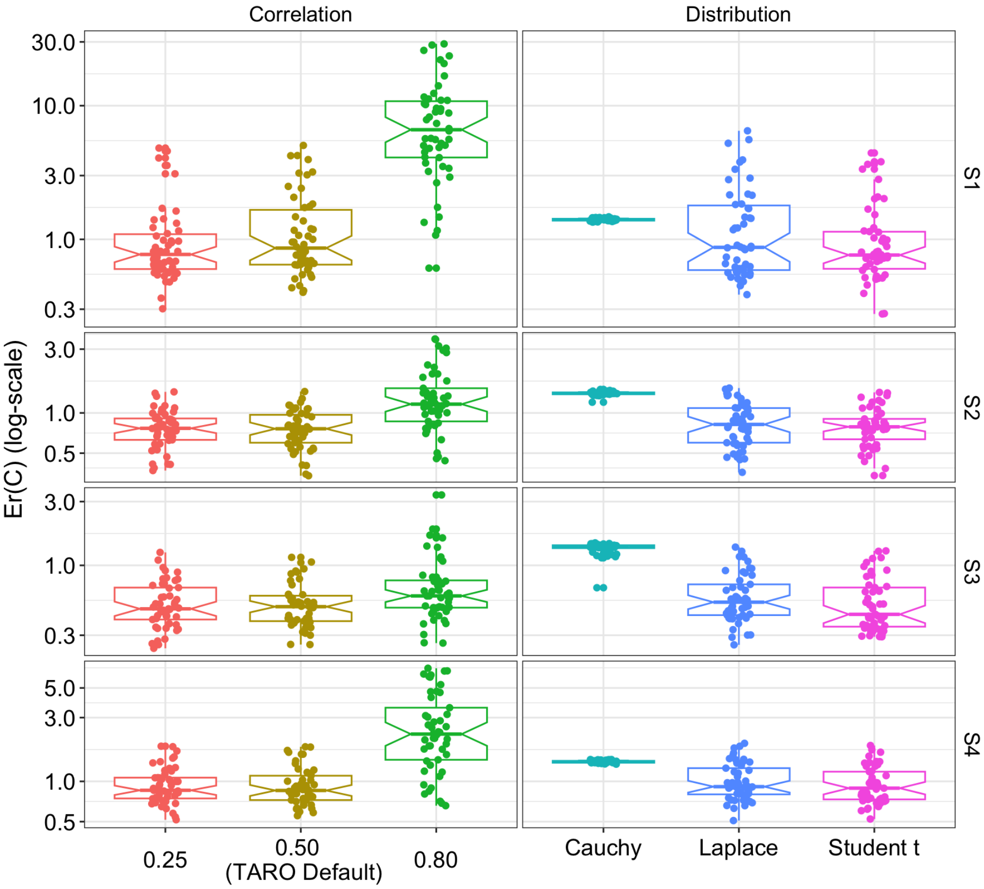

Figure S4: Simulation study: Evaluation of TARO in the presence of correlated errors among the multivariate responses and errors from heavy-tailed distributions like Cauchy, Laplace and Student's t.

S3.1.3 Evaluation of TARO with unobserved true abundance

TARO primarily operates under the assumption that even though true microbial abundance data are not directly observed, the microbial abundance data are compositionally accurate. In this subsection, we consider a simulation framework that contrasts the performance of TARO under the simulation design discussed in Section 3.1 of the main manuscript, where the observed abundances are used to generate $\mathbf{Y}$ and also as input to TARO, with a scenario where the true unobserved relative abundances are used to generate $\mathbf{Y}$, while the observed abundances are used as inputs to TARO. To construct the observed abundances from the true unobserved relative abundances, we sample from a multinomial distribution with the the sequencing depth as the number of trials and the true relative abundances as the event probabilities. We summarize the results in Figure S5 for the four settings. Our simulation study suggests that TARO's performance remains comparable to the scenario considered in the main manuscript.

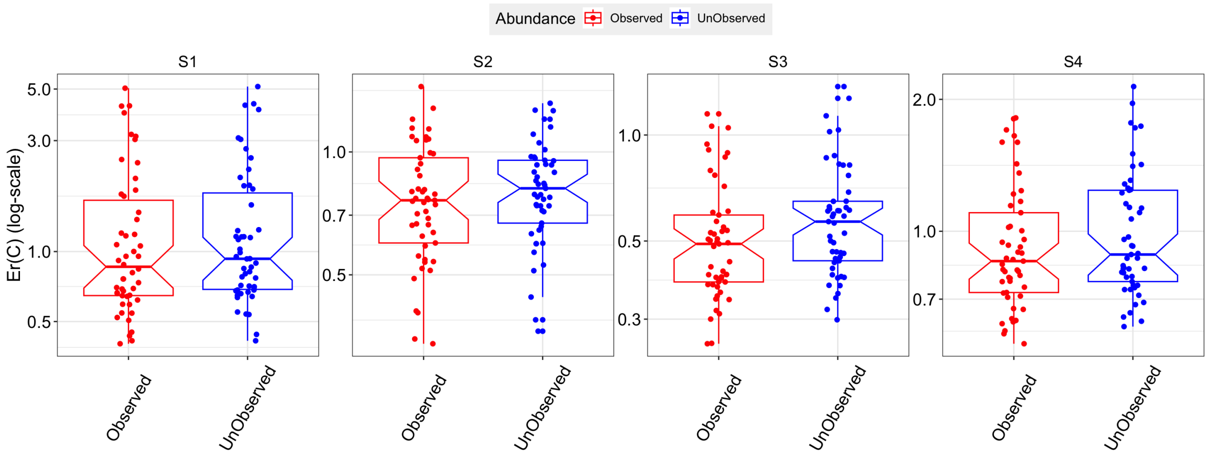

Figure S5: Simulation study: Evaluation of TARO in the four settings with observed and unobserved microbial abundance data.

S3.2 Application results

We provide a snapshot of the logistic regression modeling results in the upper left of Figure S6. Rectangular heatmaps showing the contribution of input features to the clinically relevant latent factors are shown for the metabolite variables (upper right) and microbiome features (bottom). We observed that the microbiome features contributing to the clinically relevant latent factors were at the family and genus level: X2 includes 8 family-level and 68 genus-level features, X5 includes 6 family-level and 75 genus-level features, and X7 includes 4 family-level and 76 genus-level features. Across the eight latent factors inferred by TARO, aggregation to higher tree levels was relatively rare, with 1 phylum, 2 classes, and 6 orders selected in total across all latent factors. The results of metabolite set enrichment analysis for the clinically relevant latent factors are shown in Figure S7.

References

T. Goldstein and S. Osher. The split bregman method for l1-regularized problems. *SIAM J. Img. Sci.*, 2(2):323–343, Apr. 2009. ISSN 1936-4954. doi: 10.1137/080725891.

A. Mishra, D. K. Dey, Y. Chen, and K. Chen. Generalized co-sparse factor regression. *Computational Statistics & Data Analysis*, 157:107127, 2021.

H. Zou and T. J. Hastie. Regularization and variable selection via the elastic net. *Journal of the Royal Statistical Society: Series B (Statistical Methodology)*, 67(2):301–320, 2005. ISSN 1467-9868.

H. Zou and H. H. Zhang. On the adaptive elastic-net with a diverging number of parameters. *Annals of Statistics*, 37(4):1733, 2009.

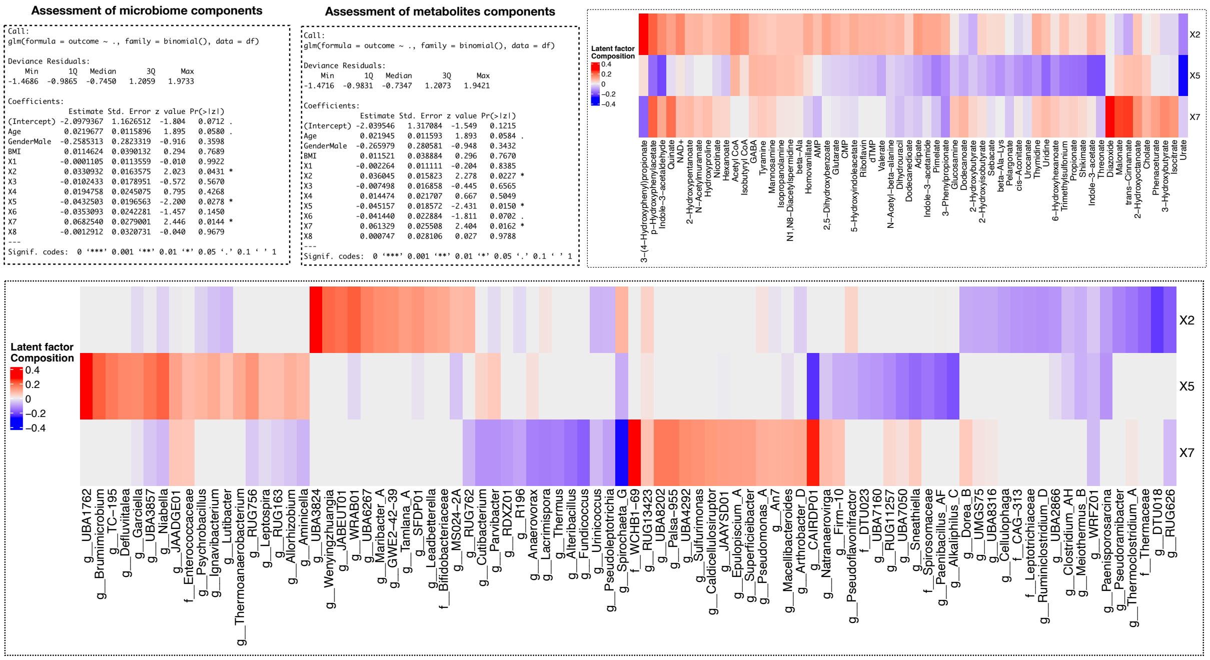

Figure S6: Assessment of the eight microbiome/metabolites latent factors in association with the outcome of interest (healthy vs colorectal cancer patients). Heatmaps show the selected microbiome and metabolites in respective latent factors.

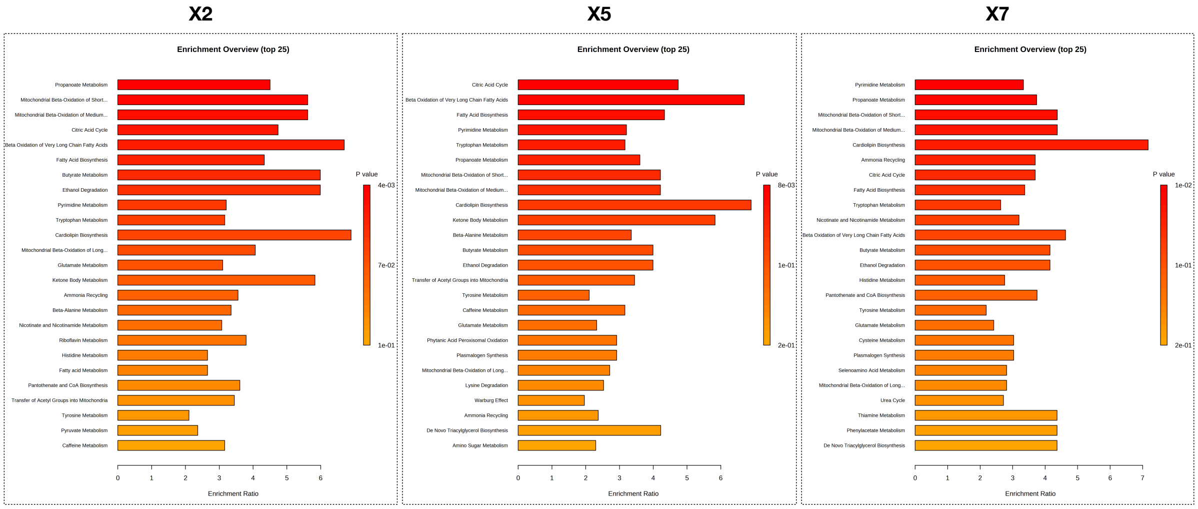

Figure S7: Metabolic pathway obtained using metabolite set enrichment analysis.
